# Metabolic Perturbations from Step Reduction in Older Persons at Risk for Sarcopenia: Plasma Biomarkers of Abrupt Changes in Physical Activity

**DOI:** 10.3390/metabo9070134

**Published:** 2019-07-08

**Authors:** Michelle Saoi, Alice Li, Chris McGlory, Tanner Stokes, Mark T. von Allmen, Stuart M. Phillips, Philip Britz-McKibbin

**Affiliations:** 1 Department of Chemistry and Chemical Biology, McMaster University, Hamilton, ON L8S 4M1, Canada; 2 Department of Kinesiology, McMaster University, Hamilton, ON L8S 4K1, Canada

**Keywords:** metabolomics, capillary electrophoresis, mass spectrometry, sarcopenia, ageing, obesity, physical inactivity

## Abstract

Sarcopenia is the age-related loss of skeletal muscle mass, strength and function, which may be accelerated during periods of physical inactivity. Declines in skeletal muscle and functionality not only impacts mobility but also increases chronic disease risk, such as type 2 diabetes. The aim of this study was to measure adaptive metabolic responses to acute changes in habitual activity in a cohort of overweight, pre-diabetic older adults (age = 69 ± 4 years; BMI = 27 ± 4 kg/m^2^, *n* = 17) when using non-targeted metabolite profiling by multisegment injection-capillary electrophoresis-mass spectrometry. Participants completed two weeks of step reduction (<1000 steps/day) followed by a two week recovery period, where fasting plasma samples were collected at three time intervals at baseline, after step reduction and following recovery. Two weeks of step reduction elicited increases in circulatory metabolites associated with a decline in muscle energy metabolism and protein degradation, including glutamine, carnitine and creatine (*q* < 0.05; effect size > 0.30), as well as methionine and deoxycarnitine (*p* < 0.05; effect size ≈ 0.20) as compared to baseline. Similarly, decreases in uremic toxins in plasma that promote muscle inflammation, indoxyl sulfate and hippuric acid, as well as oxoproline, a precursor used for intramuscular glutathione recycling, were also associated with physical inactivity (*p* < 0.05; effect size > 0.20). Our results indicate that older persons are susceptible to metabolic perturbations due to short-term step reduction that were not fully reversible with resumption of normal ambulatory activity over the same time period. These plasma biomarkers may enable early detection of inactivity-induced metabolic dysregulation in older persons at risk for sarcopenia not readily measured by current imaging techniques or muscle function tests, which is required for the design of therapeutic interventions to counter these deleterious changes in support of healthy ageing.

## 1. Introduction

Sarcopenia is the progressive, degenerative decline in skeletal muscle mass, function and strength and is measurable after the fifth decade of life [1,2,3] affecting 5–13% of 60–70 year olds and up to 50% of those aged 80 years and older [4]. Multiple factors contribute to sarcopenia, including age-related biological changes (e.g., chronic inflammation, oxidative stress, hormonal alterations), malnutrition (e.g., decreased protein and total caloric intake), as well as physical inactivity [1,4,5]. All these stressors contribute to profound physiological and morphological changes in skeletal muscle structure and function, leading to a loss in functionality and independence [2,6] with greater physical frailty [7]. Increasing rates of obesity have also been implicated in the higher incidence of sarcopenia, with older adults faced with the combined metabolic burdens of excess adiposity and reduced muscle mass referred to as sarcopenic obesity [8]. Nevertheless, a consensus on an exact definition of sarcopenia remains elusive due to comorbidity with other diseases, as well as inconsistent thresholds, reference standards and instrumental methods used for measuring lean body mass despite its recent recognition as an independent condition in 2016 [9].

The current gold-standard for diagnosis of sarcopenia relies on imaging techniques based on dual-energy X-ray absorptiometry (DXA) that provides an index of skeletal muscle mass [10]; however inaccuracies have been reported when comparing data from different manufacturers/instruments [11] and sensitivity is limited when detecting small changes in muscle mass for early detection of sarcopenia. For these reasons, qualitative measures of muscle strength and function are also assessed using standardized performance tests such as the handgrip strength test [12], gait speed [13] and short physical performance battery (i.e., balance, walking speed and strength) [14]. Thus, both functional and quantitative measures of skeletal muscle health are needed to better distinguish sarcopenia from other muscle-related ageing processes prevalent in older adults since gains in muscle mass do not necessarily prevent age-related loss in muscle strength [15]. The progressive loss in muscle quality, strength and metabolic decline contributes to a profound decline in quality of life in affected older persons with deleterious health outcomes, including physical impairments, chronic disease risk (e.g., type 2 diabetes), as well as cognitive impairments and depression [2,6]. For these reasons, sarcopenia places an increasingly severe burden on public healthcare resources due to loss of mobility, with greater risk for falls or fractures resulting in new or prolonged hospitalization and institutionalization [16,17]. Direct and indirect healthcare expenditures associated with sarcopenia are projected to expand dramatically due to an ageing demographic in most developed countries [5].

As a result, there is an urgent need to implement public policies that promote healthy ageing to prevent sarcopenia on a population level; however, a fundamental understanding of the molecular mechanisms associated with early onset sarcopenia is lacking, including reliable screening tools for identifying high risk individuals. Herein, we performed non-targeted metabolite profiling using multisegment injection-capillary electrophoresis-mass spectrometry (MSI-CE-MS) on fasting plasma samples collected from a cohort of older adults who completed two weeks of reduced daily stepping, followed by two weeks of recovery upon return to habitual ambulatory activity [18]. We aimed to elucidate adaptive metabolic responses to abrupt changes in physical activity (via step reduction), an increasingly common scenario in ageing, based on dynamic changes in the plasma metabolome at three time intervals during the intervention. Our work identified a panel of circulating biomarkers that reflect inactivity-induced metabolic dysregulation corresponding to early stages of protein degradation within muscle tissue. To the best of our knowledge, this is the first metabolomics study to examine the systemic effects from physical inactivity on overweight/pre-diabetic older persons at risk for sarcopenia, which was used as a model system of muscle disuse less severe than prolonged bed-rest or hospitalization cases.

## 2. Results

### 2.1. High Throughput Metabolomic Studies of Plasma Filtrates Using MSI-CE-MS

**Figure 1:**
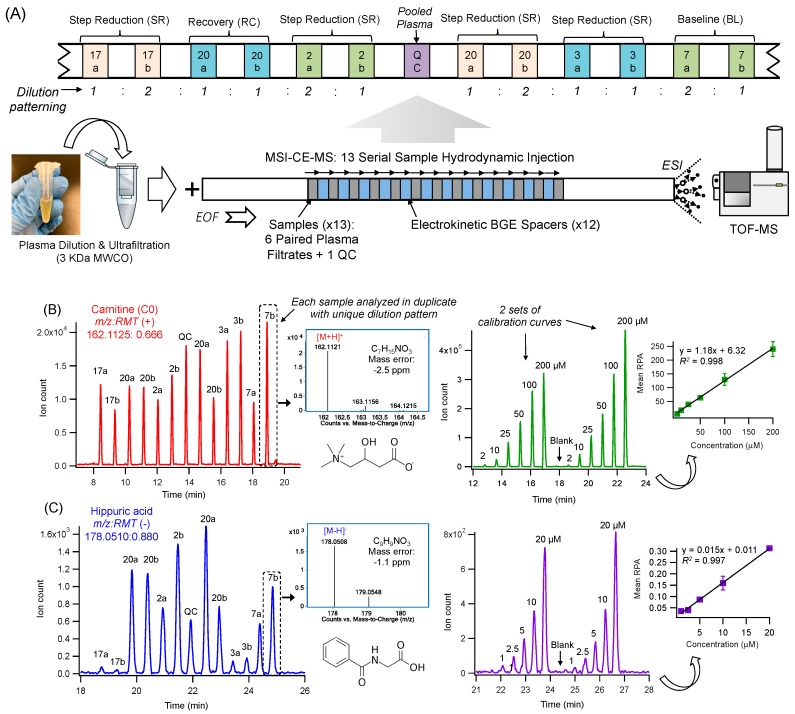
Multiplexed separations by multi-segment injection-capillary electrophoresis-mass spectrometry (MSI-CE-MS) for rapid metabolite profiling of 13 samples within a single run, including their reliable identification and quantification in plasma. (**A**) Plasma filtrates were prepared after ultrafiltration and then randomly analyzed as paired duplicate samples using a temporal dilution pattern (1:2, 1:1, 2:1) at three time points in this repeated measures step reduction intervention. Also, a pooled plasma quality control (QC) and/or blank filtrate sample was injected within each in run in order to assess technical precision and sample carry-over effects, respectively. (**B**) Representative extracted ion electropherograms for a cationic plasma metabolite, carnitine as denoted by its *m/z*:RMT (162.112:0.666, [M+H]^+^) and most likely molecular formula with low mass error. Two different serial injection configurations in MSI-CE-MS are depicted here, including a randomized analysis of six pairs of plasma samples from each participant at a specific time point from this study and a duplicate six-point calibration curve for carnitine quantification. (**C**) Two analogous extracted ion electropherograms are shown for a major anionic plasma metabolite detected under negative ion mode, hippuric acid as denoted by its *m/z*:RMT (178.0510:0.880, [M-H]^−^) and most likely molecular formula with low mass error. Note the much larger between-subject biological variance of plasma hippuric acid relative to circulating levels of carnitine.

Multiplexed separations based on MSI-CE-MS offer a high throughput platform for biomarker discovery in metabolomics that is optimal for analysis of mass or volume-restricted biological specimens ranging from dried blood spot punches, infant sweat specimens to skeletal muscle tissue biopsies [19,20,21,22]. Major improvements in sample throughput are achieved without added infrastructure costs or complicated column switching programs, which allows for stringent quality control (QC) and batch correction [23]. To date, previous studies have employed a serial hydrodynamic injection of discrete sample plugs (i.e., typically seven) between alternating segments of background electrolyte (BGE) within a single experimental run [24]. In this work, we implemented a modified serial injection format in MSI-CE-MS, comprising of 13 plasma filtrate samples when using electrokinetic BGE spacers in order to further boost sample throughput (<3 min/sample), separation resolution and peak capacity as compared to conventional single injection separations. This process initiates zonal electrophoretic separation of ions and their isomers/isobars immediately after each sample injection and thus, takes advantage of the total effective capillary length as shown in Figure 1A. Novel experimental workflows can be designed in MSI-CE-MS via customized, serial injection configurations that is effective for metabolite authentication when performing non-targeted profiling in order to reject a plethora of background or redundant signals (e.g., in-source fragments, isotope signals, adduct ions) generated in ESI-MS [20,25]. For example, a randomized injection of plasma filtrate samples from each participant was analyzed in duplicate with a distinct dilution pattern (1:2, 1:1, 2:1) to encode sample position information reflecting the study design of the experiment (Figure 1B). Each run in MSI-CE-MS also includes a pooled QC and/or blank filtrate to assess technical precision and potential sample carry-over between injections, respectively. Each ion was annotated based on its characteristic accurate mass and relative migration time (*m/z*:RMT) under positive (+) or negative (-) ion mode detection with putative identification (level 2) based on its most likely molecular formula using high resolution MS as shown in extracted ion electropherograms for representative cationic (Figure 1B) and anionic (Figure 1C) plasma metabolites, such as carnitine (162.112:0.666 as [M+H]^+^) and hippuric acid (178.051:0.880 as [M-H]^−^). Unambiguous identification of plasma metabolites (level 1) was then achieved based on their co-migration and MS/MS spectral match when compared to an authentic standard based on recommended guidelines from the Metabolomics Standards Initiative [26,27]. Once identified, a different injection format in MSI-CE-MS was used to acquire calibration curves for metabolite quantification in plasma samples as shown for a series of calibrant solutions for carnitine and hippuric acid under positive and negative ion modes, respectively (Figure 1B,C).

### 2.2. Plasma Metabolic Phenotyping of Healthy Seniors in a Step Reduction Study

This work involved non-targeted metabolite profiling of plasma samples collected from a cohort of overweight, pre-diabetic older men and women participating in a step reduction trial (ClinicalTrials.gov #NCT03039556) [18]. In order to minimize confounding effects, all participants were provided standardized meals for 3 days prior to clinical visits to collect fasting plasma in the morning while maintaining normal habitual diet/activity with exception of the two-week step reduction period (<1000 steps/day). Only participants (*n* = 17 of 22) who had complete fasting plasma samples collected at three time intervals were included in this study [18], which included slightly more males (58%) with a mean age of 69 years who were largely overweight Caucasians with a mean BMI of 27 kg/m^2^ recruited from the local community as summarized in Table 1. Overall, all participants underwent about an eight-fold reduction (*p* = 2.70 × 10^−6^) in habitual physical activity via step reduction as compared to baseline and recovery periods as confirmed by a pedometer assigned to each participant, as well as independent armband measurements [18]. As expected, this drastic change in habitual activity resulted in a corresponding decrease in total energy expenditure (*p* = 2.99 × 10^−4^) and a modest reduction in myofibrillar protein biosynthesis (*p* = 0.040) following step reduction [18]. All participants were generally healthy and moderately active upon recruitment for this study; however, standardized oral glucose tolerance tests (OGTT) during clinical visits revealed that a majority (53%) of participants were at risk for pre-diabetes at baseline due to impaired fasting glucose (6.1–6.9 mM) and/or impaired glucose tolerance (2hPG = 7.8–11 mM) based on diagnostic criteria defined by Diabetes Canada [28]. After completing step reduction and recovery periods, three additional subjects had 2hPG levels that satisfied criteria for pre-diabetes. Therefore, after the intervention about 88% of the study cohort were defined as pre-diabetic who are at risk for cardiovascular disease [29], with three (23%) subjects at highest risk since they had both impaired fasting glucose and impaired glucose tolerance. Supplemental Figure S1 depicts individual changes in glucose metabolism for all participants in this study, which also highlights that one participant with borderline impaired fasting glucose at baseline later developed diabetes after step reduction. Indeed, step reduction resulted in a modest overall decrease in glucose tolerance (*p* = 0.070) in this cohort of largely pre-diabetic, overweight older persons.

**Figure 2:**
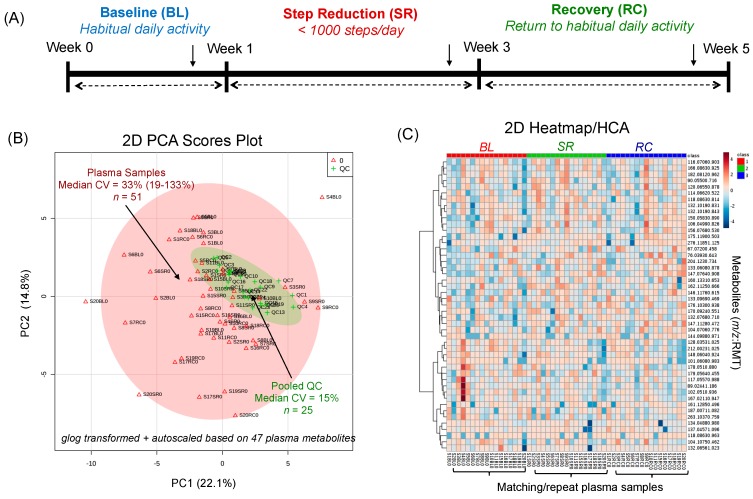

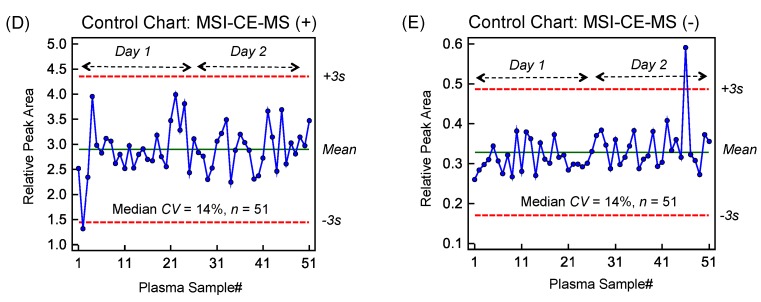
(**A**) Study design of this repeat measures intervention trial with fasting plasma samples collected from a cohort of older, pre-diabetic adults at three time intervals, including baseline (BL), after 2 weeks of step reduction (SR) and following 2 weeks of recovery to habitual physical activity (RC). Two unsupervised multivariate data analysis methods for summarizing *glog*-transformed and autoscaled plasma metabolome data, including a (**B**) 2D scores plot from PCA used to compare the biological variance of the plasma metabolome between-subjects relative to the technical precision from repeat analysis of pooled plasma as QC and a (**C**) 2D heat map with HCA used to depict the overall data structure in this study. Also, control charts for recovery standard (F-Phe) measured under (**D**) positive ion and (E) negative ion modes for all plasma samples demonstrate acceptable intermediate precision (CV = 14%) with few outliers (2 out of 102) exceeding warning limits (±3 s).

Figure 2A depicts the overall study design which included repeat fasting plasma samples collected at three time intervals for each participant at baseline, after two weeks of step reduction and following recovery when resuming normal ambulatory activity for another two week period. Stringent selection criteria were applied when performing nontargeted metabolite profiling of plasma in order to reduce bias and false discoveries as described elsewhere [20,21]. Briefly, after initial metabolite authentication and identification when using a dilution trend filter on a pooled plasma sample, only frequently detected plasma metabolites measured in the majority of samples in this cohort (>75%) with acceptable technical precision based on repeated analysis of QCs (mean CV < 30%, *n* = 25) were included in the final data matrix. Overall, a total of 47 polar/ionic metabolites from plasma satisfied these selection criteria with most metabolites identified with authentic standards (level 1), including circulating amino acids, acylcarnitines, biogenic amines, organic acids and various secondary metabolites as their glucuronide, glycine or sulfate conjugates (Table S1 of Supporting Information). As expected, the biological variance of the plasma metabolome was considerably larger (median CV = 33%, *n* = 51) as compared to the technical precision of QCs (median CV = 15%, *n* = 25) as shown in the 2D scores plots from a principal component analysis (PCA) in Figure 2B. A 2D heat map with hierarchical cluster analysis (HCA) summarizes the overall data structure involving 47 plasma metabolites denoted by their *m/z*:RMT consistently measured in the majority of participants at three time points in this study. In all cases, average ion responses for metabolites in MSI-CE-MS were normalized to an internal standard from the same injection position to correct for variations in injection volume on-capillary [22]. Also, control charts for the recovery standard (10 μM 4-fluorophenylalanine, F-Phe) added to all thawed plasma samples prior to ultrafiltration further confirm acceptable intermediate precision (median CV = 14%, *n* = 51) based on its normalized ion response measured in (D) positive ion mode using 3-chlorotyrosine (Cl-Tyr) and (E) negative ion mode using naphthalene monosulfonic acid (NMS) as internal standards with few outliers that exceeded warning limits. As there was no evidence of long-term signal drift or systematic error, application of a QC-based batch correction algorithm was not deemed necessary unlike long-term/intermittent studies performed on shared instrumentation following service repairs and/or relocation [30]. As a result, MSI-CE-MS offers a rapid metabolomics platform for reliable metabolite quantification when relying on customized serial injections that encode mass spectral information temporally in a separation with stringent QC.

### 2.3. Evaluating the Effects Short-Term/Acute Physical Inactivity in Older Pre-Diabetic Adults

**Figure 3:**
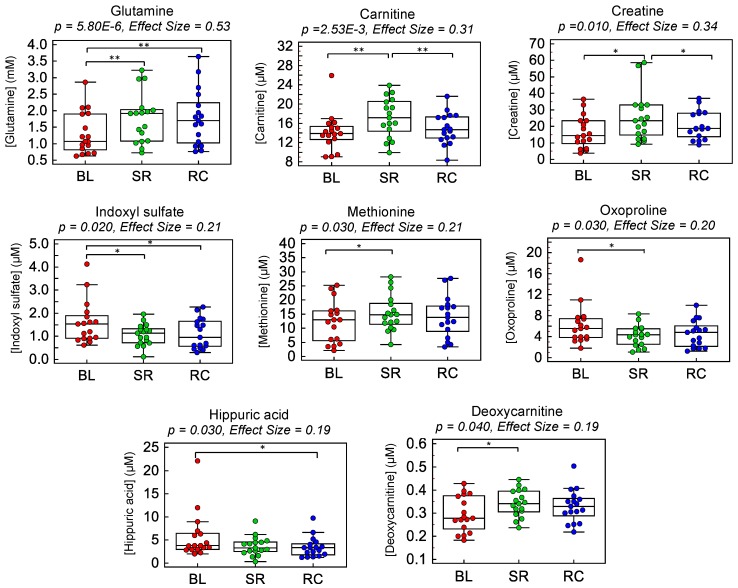
Box-whisker plots illustrating dynamic changes among eight top-ranked plasma metabolites modulated after two weeks of step reduction (SR) with a subsequent two week recovery (RC) period to normal physical activity as compared to baseline (BL) in a cohort of older pre-diabetic adults (*n* = 17). A repeated measures one-way ANOVA test was performed to identify significant changes in circulating metabolite concentrations as summarized in Table 2, where a bracket represents a significant pairwise comparison (* *p* < 0.05; ** *q* < 0.05). Overall, these plasma metabolites reflect adaptive metabolic responses to physical inactivity/muscle disuse from step reduction that did not fully recover after resuming normal habitual physical activity.

The major goal of this work was to identify dynamic plasma metabolite signatures modulated by acute step reduction and subsequent recovery to normal habitual physical activity. Overall, eight plasma metabolites were found to undergo significant changes in circulation throughout the intervention period (*p* < 0.05; effect sizes > 0.20) when using a repeat measures one-way ANOVA as summarized in Table 2. Plasma glutamine and carnitine increased after step reduction without returning back to baseline after the recovery period that satisfied a False Discovery Rate (FDR) adjustment for multiple hypothesis testing (*q* < 0.05). Additionally, plasma creatine, methionine and deoxycarnitine were also found to undergo analogous metabolic trajectories over time (*p* < 0.05). In contrast, three other plasma metabolites displayed an opposite trend with a significant decrease in circulation after step reduction (*p* < 0.05) as compared to baseline or recovery time intervals, including indoxyl sulfate, hippuric acid and oxoproline. Additionally, several ratiometric markers among the top-ranked plasma metabolites were found to increase the effect size (>0.30) and the statistical significance (*q* < 0.05, FDR) for most single plasma metabolites with the exception of glutamine as depicted in Supplemental Figure S2. As shown in the box-whisker plots in Figure 3, plasma glutamine, carnitine, creatine, methionine and deoxycarnitine showed similar trends with increasing circulating concentrations following step reduction yet remained persistently elevated after recovery when participants resumed normal ambulatory activity for two weeks. In contrast, plasma indoxyl sulfate, hippuric acid and oxoproline showed distinct decreases in circulation following step reduction relative to baseline. Nevertheless, there were large between-subject variations in plasma metabolite concentrations at baseline and variable responses to the step reduction trial when plotting individual metabolic trajectories among participants as shown in Figure S3 of the Supporting Information. For instance, while most subjects showed a general trend of increasing plasma carnitine following two weeks of step reduction, subject#8 (i.e., a pre-diabetic/overweight male) showed an opposing trend with a decreasing carnitine trajectory over time with elevated baseline concentrations. Similarly, subject#4 (i.e., a pre-diabetic/lean male) had unusually elevated baseline plasma concentrations of indoxyl sulfate, hippuric acid and oxoproline as compared to all other participants. This extent of biological variance exists even though participants were supplied with standardized meals for 3 days prior to clinical visits for blood collection while fasting. All plasma metabolites were adjusted for sex; however the extent of the step reduction was not different between subjects as a potential confounder. Additionally, a correlation matrix among the eight top-ranked plasma metabolites associated with abrupt changes in physical activity from step reduction is shown in Supplemental Figure S4, which reveals strong co-linearity between circulating levels of indoxyl sulfate and oxoproline (*r* = 0.881; *p* = 1.0 × 10^−15^), hippuric acid and oxoproline (*r* = 0.622; *p* = 1.1 × 10^−6^), hippuric acid and indoxylsulfate (*r* = 0.507; *p* = 1.4 × 10^−4^), as well as inverse correlations between glutamine and oxoproline (*r* = −0.502; *p* = 1.8 × 10^−4^) and glutamine and indoxylsulfate (*r* = −0.500; *p* = 1.8 × 10^−4^).

## 3. Discussion

Non-targeted metabolite profiling was performed on repeat plasma samples from a cohort of overweight, pre-diabetic older adults who completed two weeks of step reduction (<1000 steps/day) followed by a two week recovery period resuming their normal habitual activity. Although all participants were generally healthy and moderately active, most were pre-diabetic (88%) following step reduction and therefore susceptible in developing type 2 diabetes; however only one participant (subject#19) with borderline aberrant glucose homeostasis at baseline transitioned to diabetes as shown in Supplemental Figure S1. Overall, all participants significantly reduced their ambulatory activity and energy expenditure over a two week period (Table 1). To the best of our knowledge, this is the first metabolomics study exploring adaptive metabolic responses to an abrupt change in physical activity involving older adults. Previous results from this cohort reported lower rates of myofibrillar protein biosynthesis using a deuterated water ingestion method, as well as a worsening of glycemic control with a modest elevation in circulatory inflammatory markers (e.g., C-reactive protein, IL-6, TNF-α) following step reduction [18]; however, there was no detectable reduction in DXA-measured fat free mass, BMI or muscle strength indicative of early stages of muscle protein degradation without measurable evidence of sarcopenia (i.e., pre-clinical). Previous bed-rest studies have reported substantial muscle atrophy and whole-body insulin resistance likely due to a more pronounced reduction in daily physical activity [31,32] as compared to this study. Similarly, a single day of bed rest was not reported to induce any changes in skeletal muscle deconditioning or gene expression associated with the regulation of muscle mass and insulin sensitivity [33]. In contrast, changes in insulin sensitivity was reported in young men following 2 weeks of step reduction when combined with overfeeding [34]. In our case, participants were requested to maintain habitual dietary patterns during the intervention with the exception of standardized meals consumed for 3 days prior to clinical visits.

Similar to previous targeted metabolomics studies which have focused on sarcopenic patients exhibiting poor muscle quality [35,36,37], several circulating metabolites closely associated with skeletal muscle metabolism were modulated by step reduction in this work. MSI-CE-MS was used as a high throughput metabolomics platform that takes advantage of customized serial injection configurations for analysis of 13 samples within a single run, including six pairs of plasma filtrates together with a pooled sample as a QC and/or blank filtrate (Figure 1). Overall, 47 plasma metabolites (Supplemental Table S1) were consistently detected in the majority of plasma samples with acceptable technical precision from a cohort of older adults participating in a repeated measures intervention trial (Figure 2). However, only eight plasma metabolites were found to undergo significant changes (*p* < 0.05) following physical inactivity via step reduction. These results are complementary to independent measurements of myofibrillar protein and genes encoding several mitochondrial protein within muscle tissue [18]. In this work, plasma glutamine was by far the most significant biomarker associated with physical inactivity that increased 1.30-fold following step reduction (*q* = 3.02 × 10^−4^; effect size = 0.53), which was independent of sex and extent of step reduction as potential confounders. In fact, plasma glutamine concentrations remained persistently elevated and did not fully return back to baseline after a two week recovery period to normal activity. These findings coincide with a recent report demonstrating that increases in plasma glutamine were associated with clinically diagnosed sarcopenic and community-dwelling and institutionalized elderly [37]. Glutamine is the most abundant amino acid in circulation [22] that is conditionally essential during periods of acute metabolic stress, and it is largely derived from *de novo* synthesis within skeletal muscle from glutamic acid and ammonia via glutamine synthetase [38,39]. While glutamine is involved in a myriad of metabolic pathways in the body (i.e., nitrogen transport, signal transduction, energy metabolism etc.) [38], it plays a crucial role in regulating protein synthesis and breakdown within skeletal muscle, where glutamine is used for transporting nitrogen to the liver [40]. Indeed, decreasing concentrations of intramuscular glutamine accelerate protein catabolism resulting in a decline in skeletal muscle mass which is a hallmark of sarcopenia [38,41]. Therefore, the apparent increase in plasma glutamine from step reduction could arise from greater efflux of glutamine from skeletal muscle stores into circulation indicative of a catabolic physiological state developing among older adults that is consistent with a modest decline in myofibrillar protein synthesis.

Adaptive changes in muscle energy metabolism from acute physical inactivity also plays a major role in 1.21-fold increase in plasma carnitine concentrations following step reduction (*q* = 0.020; effect size = 0.31). While carnitine may be derived from the diet and/or endogenously synthesized in the liver, kidney and brain from trimethyllysine [42], plasma carnitine is a major source for skeletal muscle carnitine and subsequent synthesis of intracellular acylcarnitines for fatty acid β-oxidation. Thus, in the absence of changes in habitual diet during the intervention, our findings support that increased plasma carnitine concentrations with step reduction may be caused by its decreased uptake within skeletal muscle due to fewer demands for muscle contractions with prolonged inactivity that triggers an increase in protein catabolism and a decline in mitochondrial biogenesis. Similarly, deoxycarnitine (or γ-butyrobetaine) showed a modest 1.16-fold increase in plasma concentrations as a result of step reduction (*p* = 0.044; effect size = 0.19). Deoxycarnitine is a known precursor of carnitine that undergoes hydroxylation via γ-butyrobetaine hydrolase (BBOX) in the liver and/or kidneys to form carnitine [43]. Thereafter, deoxycarnitine is released in circulation in order to be taken up by skeletal muscle for storage. Our results confirmed an increase in circulatory deoxycarnitine following step reduction, which was likely caused by a reduced uptake capacity within muscle tissue.

Similar to the trends identified in metabolite trajectories for glutamine, carnitine and deoxycarnitine in plasma, two weeks of step reduction also resulted in a 1.57-fold increase in plasma creatine concentrations (*p* = 0.010; effect size = 0.34) as shown in Figure 3. Creatine is primarily synthesized in the kidneys and liver, subsequently excreted into circulation and further stored within skeletal muscle, where it plays important roles in energy metabolism and muscle function [44,45] as it is transformed into the high energy phosphate donor, phosphocreatine to regenerate ATP during active muscle contractions with strenuous exercise [22]. However, prolonged periods of physical inactivity have shown to severely decrease intramuscular creatine concentrations due to muscle atrophy and protein catabolism. For instance, MacDougall et al. [46] reported a 25% reduction in muscle phosphocreatine in healthy, recreationally active men after five weeks of experimentally-induced immobilization. Similar declines may be paralleled in older adults with sedentary lifestyles, including those that are hospital-bound and bed-ridden. As a result, increases in plasma creatine concentrations likely mirror a corresponding decrease in intramuscular creatine with greater muscle protein turn-over rates after two weeks of step reduction. A recent study by Garvey et al. [47] also reported increased plasma creatine levels and decreased muscle creatine in aged rats, which was attributed to skeletal muscle “leakage,” promoting the loss of creatine from skeletal muscle stores due to age-related declines in muscle mass.

Step reduction involving older adults also elicited a 1.20-fold increase in plasma methionine levels (*p* = 0.028; effect size = 0.21) as compared to baseline. Our findings coincided with a previous study by Moaddel et al. [36], concluding that increased circulating methionine levels were associated with low muscle quality in older persons. Methionine is an essential sulfur-containing amino acid that plays crucial roles in several metabolic processes (e.g., polyamine, creatine and phosphatidylcholine metabolism), as well as protein synthesis and redox homeostasis [48]. Methionine is also an immediate precursor of cysteine, a rate-limiting substrate for intracellular glutathione biosynthesis that is upregulated during oxidative stress, a hallmark of ageing and sarcopenic obesity who have higher circulatory levels of oxidized glutathione than non-sarcopenic controls [49]. Indeed, glutathione biosynthesis activity and intracellular glutathione concentrations both tend to decline with ageing [50], which is exacerbated by low dietary protein and/or inadequate sulfur amino acid intake common among older persons [51]. Interestingly, our study also revealed several unexpected plasma biomarkers of physical inactivity as a result of step reduction, including indoxyl sulfate (*p* = 0.021) and hippuric acid (*p* = 0.033), as well as oxoproline (*p* = 0.025) with similar modest effect sizes (≈0.20). The former two plasma metabolites function as uremic toxins that often accumulate in plasma due to chronic kidney disease (CKD) prevalent among patients with type 2 diabetes, which have been shown to contribute to deleterious health effects by stimulating muscle atrophy, protein catabolism and CKD-induced sarcopenia (i.e., uremic sarcopenia) [52,53]. Increased intramuscular indoxyl sulfate has also recently been reported to induce metabolic alterations in skeletal muscle tissue that increase oxidative stress and triggers inflammatory cytokines (e.g., TNF-α, IL-6) with higher expression of myostatin and atrogin-1, leading to inhibition of muscle growth and development, and muscle atrophy [53]. We hypothesize that the decline in plasma indoxyl sulfate following step reduction may be a result of its increased uptake within skeletal muscle that promotes muscle wasting in susceptible older adults. The exact biochemical role of hippuric acid in step reduction/muscle disuse is not clear but both indoxyl sulfate and hippuric acid are major protein-bound uremic toxins [54] that are modulated by dietary intake, host liver metabolism and gut microflora as reflected by their co-linearity in fasting plasma samples (*r* = 0.507; *p* < 1.4 × 10^−4^, Supplemental Figure S4). Our study also revealed a corresponding 0.67-fold lowering of plasma oxoproline (or pyroglutamic acid), a known precursor of intracellular glutathione, produced through the γ-glutamyl dipeptide cycle that is a key glutathione salvage pathway activated during oxidative stress [55,56]. The adaptive decrease in circulating oxoproline concentrations after step reduction suggests its higher intramuscular uptake to promote glutathione biosynthesis in order to combat increases in reactive oxygen species (ROS) from muscle disuse with step reduction. Interestingly, plasma oxoproline was strongly correlated to both indoxyl sulfate (*r* = 0.881; *p* < 1.0 × 10^−15^) and hippuric acid (*r* = 0.622; *p* < 1.1 × 10^−6^) implying a direct coupling of intra-muscular oxidative stress/glutathione depletion and increased uremic toxin uptake within muscle tissue resulting in lower oxoproline in circulation. Similar to most other biomarkers of muscle disuse from step reduction (Table 2), plasma oxoproline concentrations remained persistently lower as compared to baseline even two weeks following recovery with a return to normal ambulatory activity. Muscle metabolomic studies [22] are needed to better understand the relationship between changes in circulatory metabolism with skeletal muscle health in older adults, however access to residual muscle tissue specimens was not available for analysis in this study.

**Figure 4:**
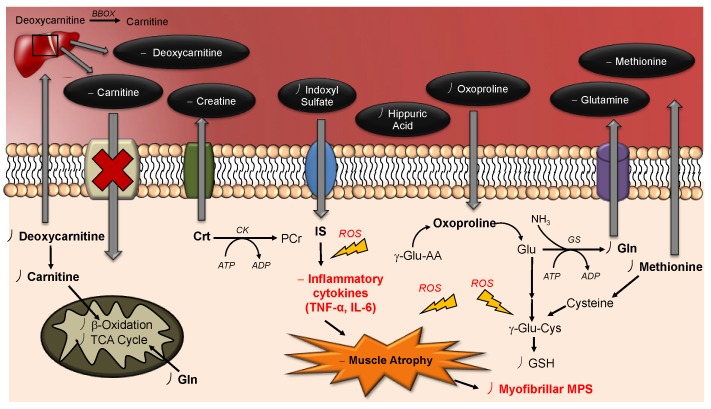
Schematic illustrating the systemic effects of step reduction in older adults on their circulatory metabolism, as well as metabolic changes within skeletal muscle. Prolonged physical inactivity elicits several metabolic and physiological adaptations in skeletal muscle, including a lower rate of myofibrillar protein synthesis that contributes to protein catabolism. Increases in plasma creatine and glutamine concentrations likely reflect early stages of muscle mass decline due to their loss from major skeletal muscle stores. Also, carnitine and deoxycarnitine concentrations accumulate in plasma following step reduction due to reduced uptake capacity within skeletal muscle contributing to a downregulation in lipid metabolism (i.e., β-oxidation) and greater risk for mitochondrial dysfunction. Step reduction also led to declines in circulating uremic toxins, indoxyl sulfate and hippuric acid indicative of their intramuscular accumulation that contributes to oxidative stress products (ROS) and inflammatory cytokines (TNF-α, IL-6) thereby promoting muscle atrophy. Greater oxidative stress within skeletal muscle also depletes intracellular glutathione given limiting amounts of cysteine or methionine. Also, plasma oxoproline, a key precursor used in glutathione recycling, decreases in circulation as it is transported within muscle to support intracellular glutathione biosynthesis in response to oxidative stress from physical inactivity. BBOX: γ-Butyrobetaine hydrolase; Crt: Creatine; CK: Creatine kinase; Gln: Glutamine; Glu: Glutamic acid; GS: Glutamine synthetase; GSH: Glutathione; IL-6: Interleukin-6; IS: Indoxyl sulfate; MPS: Muscle protein synthesis; PCr: Phosphocreatine; ROS: Reactive oxidative species; TNF-α: Tumor necrosis factor alpha; γ-Glu-AA: γ-glutamyl-amino acids.

Figure 4 depicts an illustrative scheme that provides an overview of the adaptive metabolic perturbations following step reduction that may provide new targets for therapeutic interventions in managing or preventing sarcopenia among high-risk older persons. Overall, top-ranked plasma biomarkers of muscle disuse were largely associated with changes in skeletal muscle energy metabolism (creatine, carnitine, deoxycarnitine) and/or protein catabolism (glutamine), as well as glutathione biosynthesis (methionine, oxoproline) and uremic toxins (indoxyl sulfate, hippuric acid) which are likely contributing factors to metabolic acidosis, muscle protein wasting and uremic sarcopenia. In order to ameliorate the effects of protein catabolism (i.e., muscle atrophy) due to ageing, nutritional supplementation has long been proposed to increase muscle protein synthesis and thus, increase muscle mass in geriatric populations. Interestingly, many metabolites identified in this study (e.g., glutamine, creatine and carnitine) are widely used dietary supplements for enhancing overall muscle health, strength and performance [57]. In fact, previous studies have demonstrated that high dose creatine supplementation led to improvements in muscle strength and performance in daily activities, while preventing bone loss in the older adults [58,59]. In contrast, glutamine supplementation in aged rats was reported to elicit no improvements in protein synthesis and thus, was insufficient to increase skeletal muscle mass to combat age-related muscle atrophy accelerated due to physical inactivity [60]. Similarly, oral supplementation with carnitine has not shown beneficial effects on skeletal muscle mass and function in older women [61] despite its widespread use as a nutritional supplement to support muscle energy metabolism. Alternatively, there is growing evidence that resistance training can elicit a number of metabolic adaptations that can combat the adverse effects associated with age-related declines in muscle mass and function in older persons [62,63]. These improvements can be further enhanced when coupling protein supplementation with resistance training to improve muscle mass and combat age-related muscle atrophy [64]. Future studies will validate the impact of safe yet effective lifestyle modifications based on diet and/or exercise to attenuate the deleterious effects of physical inactivity on skeletal muscle health that promotes healthy ageing.

Although this work provided novel insights to the deleterious metabolic responses to physical inactivity in high-risk older adults, there were several study limitations. Firstly, a larger and more diverse cohort of participants is needed to increase study power due to significant between-subject variance while further validating these findings in other community dwelling centers. Also, there were no measurable phenotypic changes in free fat mass, BMI or muscle strength in this study that likely require a longer intervention period of acute muscle disuse. Furthermore, access to muscle tissue biopsies is required to better interpret adaptive metabolic changes in circulatory metabolism to those occurring strictly within skeletal muscle. While this study focused on the characterization of the polar/ionic metabolites in plasma, future studies should expand metabolome coverage to include circulating fatty acids and intact lipids when using multiplexed separations with nonaqueous-CE-MS [23] given their essential roles in muscle energy metabolism and modulators of inflammation. A major finding of this study was that otherwise healthy and moderately active older persons were susceptible to metabolic stresses and catabolic processes that were not fully recoverable when resuming normal ambulatory activity, including a panel of plasma metabolites associated with muscle energy metabolism, protein breakdown and oxidative stress from glutathione depletion and inflammatory uremic toxin exposures. Additionally, these same metabolites may serve as useful biomarkers for monitoring sarcopenia progression and novel treatment interventions for its prevention. In this case, longer recovery times (>2 weeks) can be explored to determine if these metabolic changes do indeed return to baseline, which may be accelerated with optimal resistance training and/or nutritional supplementation regimes suitable for older adults. The analysis of biomarkers identified in this work from non-invasive biological fluids, such as urine may also enable routine screening for pre-symptomatic detection of sarcopenia not measurable by conventional body imaging or muscle function tests in older adults.

## 4. Materials and Methods

### 4.1. Study Cohort and Intervention

This study was based on a previously published study where 22 older adults (12 men, 10 women) were recruited to undergo an acute step reduction intervention [18]. However, only 17 (10 men, 7 women) of the 22 participants were included in our study due to lack of plasma specimens available at each time point. Prior to the intervention, participants were screened to ensure they met the inclusion criteria (i.e., non-smoking, free from chronic disease, moderately active, did not consume nonsteroidal anti-inflammatory drugs or medication for cholesterol management). The study protocol was approved by the Hamilton Integrated Research Board (REB #14-609). First, participants underwent 7 days of monitored normal physical activity (baseline; BL). Thereafter, participants underwent two weeks of step reduction (SR, <1000 steps per day) followed by return to habitual physical activity for two weeks during recovery (RC). Daily step count was monitored using a hip-placed pedometer unit (Piezo SC-StepX Health System, StepsCount, Deep River, ON, Canada), which was internally validated with a SenseWear arm band accelerometer (BodyMedia, Pittsburg, PA, USA). During the last three days of each intervention period (BL, SR and RC), participants were provided with standardized meals (55% carbohydrate (CHO), 30% fat and 15% protein) in the form of flash frozen, prepackaged foods (Heart to Home, Hamilton, ON, Canada). Furthermore, at the end of each intervention period (BL, SR and RC), participants also performed an oral glucose tolerance test (OGTT) following a 10 h overnight fast where blood samples were obtained via an intravenous catheter inserted into an antecubital vein. After collection, blood samples were centrifuged at 4000× *g* for 10 min at 4 °C. Afterwards, all plasma samples were stored at −80 °C prior to further preparation for metabolomics analysis.

### 4.2. Plasma Sample Preparation

Frozen raw plasma was slowly thawed on ice, vortexed for 30 s and aliquoted. An aliquot of 50 μL of plasma was diluted two-fold with ultra-grade LC-MS water (Caledon Laboratories Ltd., Georgetown, ON, Canada) containing 40 μM of the recovery standards, 4-fluoro-L-phenylalanine (F-Phe) and 3-cyclohexylamino-1-propanesulfonic acid (CAPS), for both positive and negative mode ESI-MS. The diluted plasma was vortexed for 30 s and transferred to a pre-rinsed 3 kDa molecular weight cutoff (MWCO) ultrafiltration tube (Pall Life Sciences, Port Washington, NY, USA) and filtered at 14,000× *g* for 7.5 min to remove proteins with the plasma filtrate used for analysis. Ultrafiltration tubes were pre-rinsed with ultra-grade LC-MS water, centrifuged for 5 min at 14,000× *g* and air dried for about 20 min prior to use. Thereafter, the plasma filtrates were obtained and aliquoted into two centrifuge tubes in order to prepare two aliquots with 1:2 ratio dilution for the temporal signal pattern recognition configuration for MSI-CE-MS analysis. For the 1-fold diluted samples, 20 μL of ultra-grade LC-MS water containing 80 μM of the internal standards, 3-chloro-L-tyrosine (Cl-Tyr) and 2-napthalenesulfonic acid (NMS) and 16 mM of ^13^C-glucose was added to 15 μL of plasma filtrate. For the 2-fold diluted samples, 30 μL of ultra-grade LC-MS water containing the internal standards and ^13^C-Glucose was added to 10 μL of plasma filtrate. The final concentrations of the recovery and internal standards were 10 μM and 2 mM for ^13^C-Glucose in the diluted plasma filtrates for MSI-CE-MS analysis.

### 4.3. Nontargeted Metabolite Profiling of Plasma Filtrates by MSI-CE-MS

Non-targeted metabolite profiling using MSI-CE-MS was performed on an Agilent 7100 capillary electrophoresis (CE) instrument (Agilent Technologies Inc., Mississauga, ON, Canada) coupled to an Agilent 6230 Time-of-Flight Mass Spectrometer (TOF-MS) equipped with a coaxial sheath liquid (Dual AJS) Jetstream electrospray ion source with heated nitrogen gas. The CE separations were performed using uncoated fused-silica capillaries (Polymicro Technologies, AZ, USA) with 50 μm inner diameter and 120 cm total length. The background electrolyte (BGE) consisted of 1 M formic acid with 15% vol acetonitrile (pH 1.80) for positive ion mode and 50 mM ammonium bicarbonate (pH 8.50) for negative ion mode, which were used for nontargeted profiling of cationic and anionic metabolites, respectively [20,21,22]. In order to minimize sample carryover between injections, as well as polymer swelling/degradation upon contact with organic and/or ammonia-based solvents, the terminal ends of the capillary were burned using a MicrosolvCE Window Maker to remove 7 mm length of polyimide coating.

The serial sample injection sequence used in MSI-CE-MS consisted of 13 discrete samples injected hydrodynamically (5 s at 100 mbar) interspaced with BGE spacers injected electrokinetically at 30 kV for 75 s for the separation of cationic metabolites and 45 s for anionic metabolites. The separations were performed under normal polarity using a pressure gradient of 2 mbar/min from 0 to 40 min, with an applied voltage of 30 kV at 25 °C. Between runs, the capillary was flushed for 15 min with BGE at 950 mbar. The sheath liquid compositions consisted of 60% methanol with 0.1% vol formic acid for positive ion mode and 50% methanol for negative ion mode. Additionally, purine and hexakis(2,2,3,3-tetrafluoropropoxy)phosphazine (HP-921) (API-TOF Reference Mass Solution Kit, Agilent Technologies) were added to the sheath liquid as reference masses to provide real-time mass calibration during data acquisition. The sheath liquid was delivered at a rate of 10 μL/min using an Agilent 1260 Infinity series Isocractic Pump equipped with a 100:1 splitter. Data acquisition was performed in full-scan mode on the TOF-MS that spanned a mass range of 50–1700 *m/z* at an acquisition rate of 500 ms/spectrum. The ESI conditions were Vcap = 2000 V, nozzle voltage = 2000 V, nebulizer gas = 10 psi, sheath gas = 3.5 L/min at 195 °C, drying gas 8 L/min at 300 °C. whereas, the MS voltage settings were fragmentor = 120 V, skimmer = 65V and Oct1 RF= 750 V. During sample injection, the Vcap, nozzle voltage and nebulizer gas were turned off to minimize electrospray suctioning effects. At the beginning of each day, the TOF-MS system was calibrated over 50–1700 *m/z* range before analysis using an Agilent tune mixture to ensure residual mass ranges did not exceed 0.30 ppm. Additionally, preventative maintenance was performed such as daily cleaning of the CE electrode and ion source with 50% *v/v* isopropanol using a lint-free cloth. Thereafter, a standard mixture and pooled QCs with blank were injected to equilibrate the CE-MS system and assess system stability prior to sample analysis. After sample analysis was completed each day, the capillary was flushed for 10 min with ultra-grade LC-MS water and air dried for 10 min. The individual plasma samples were analyzed over four consecutive days in both positive and negative ESI-MS.

### 4.4. Data Processing and Statistical Analysis

All data processing and analysis were performed using Agilent MassHunter Qualitative Analysis B.06.00 and Microsoft Excel. All metabolite responses were normalized to an internal standard. Prior to univariate statistical analysis, normality testing was performed using the Shapiro-Wilk test (*p* < 0.05) on *log*-transformed data using SPSS (IBM SPSS Statistics for Windows, Version 20.0. NY, USA). A repeated measures one-way analysis of variance (ANOVA; intervention (BL, SR, RC) as within-subjects factor) was also performed on SPSS. The Mauchly’s sphericity test was initially used to determine if the data satisfied the sphericity assumption (*p* > 0.05); in cases where sphericity was violated, a Greenhouse-Geisser correction was applied accordingly. Furthermore, sex and baseline step count were tested as covariates for all metabolites and the p-values were adjusted accordingly when the effect of sex and/or baseline step count were significant. Pairwise comparisons using the Fischer’s LSD was used for *post-hoc* analysis. To correct for multiple hypothesis testing, a false discovery rate (FDR) correction using the Benjamini-Hochberg procedure was applied. Prior to multivariate analysis, all data was generalized *log*-transformed and autoscaled. Multivariate statistical analyses including principal component analysis (PCA) and hierarchical clustering analysis (HCA) were performed using Metaboanalyst 4.0 [65].

## 5. Conclusions

In this work, two weeks of acute physical inactivity induced adaptive metabolic changes in a cohort of overweight and pre-diabetic older adults who did not fully return to baseline after resuming normal ambulatory activity. Our study demonstrated increases in plasma concentrations of several metabolites associated with muscle energy metabolism (creatine, carnitine and deoxycarnitine) and protein degradation/ammonia transport (glutamine) following step reduction and muscle disuse with decreased myofibrillar protein biosynthesis. Interestingly, a decrease in plasma uremic toxins (indoxyl sulfate and hippuric acid) as well as changes in essential precursors for glutathione biosynthesis/recycling (oxoproline, methionine) was indicative of deleterious oxidative stress and inflammation within skeletal muscle following an abrupt change in physical activity among high-risk older persons. Of these metabolites, glutamine showed the most pronounced changes and largest effect size, whereas ratiometric biomarkers were found to increase the significance for other plasma metabolites. This study revealed important metabolic pathways that advance our understanding of sarcopenia at early stages of development while identifying putative biomarkers applicable to routine screening of older persons at risk for progressive muscle mass loss and chronic metabolic diseases. With the world’s geriatric population projected to increase worldwide by 2050, preventative strategies are needed to mitigate the many socioeconomic and healthcare impacts of sarcopenia and frailty in order to improve quality of life and independence while reducing the need for intensive support services and long-term care. 

## Figures and Tables

**Table 1:** Summary of physical and clinical characteristics and daily activity of study cohort.

	Baseline (BL)	Step Reduction (SR)	Recovery (RC)	*p*-Value*
Sex	10 male, 7 female	-	-	
Age (years)	69 ± 4	-	-	
Body Mass (kg)	75 ± 14	76 ± 15	76 ± 15	0.26
BMI (kg∙m^2^)	27 ± 4	27 ± 4	27 ± 4	0.16
Daily Energy Expenditure (kJ)	9890 ± 2680	8118 ± 1350	9498 ± 2310	2.99 × 10^−4^
Myofibrillar Protein Synthesis (% per day)	1.50 ± 0.06	1.33 ± 0.05	1.32 ± 0.14	0.040
Pedometer Steps (per day)	7550 ± 3320	980 ± 84	7345 ± 3850	2.70 × 10^−6^
Armband steps (per day)	6375 ± 3560	1248 ± 850	5612 ± 3740	1.33 × 10^−4^
Fasting glucose (mM)	5.24 ± 0.61	5.31 ± 0.92	5.47 ± 0.73	0.26
(mg/dL)	94 ± 11	96 ± 17	99 ± 13	0.26
2h Post-OGTT glucose (mM)	7.6 ± 1.5	9.0 ± 2.4	8.2 ± 2.9	0.070
(mg/dL)	137 ± 26	163 ± 43	147 ± 53	0.070

* *p*-values determined using a one-way repeated measures ANOVA.

**Table 2:** Top plasma metabolites and ratiometric markers significantly perturbed due to short-term step reduction as compared to baseline and recovery periods in older adults (n = 17).

*m/z*:RMT:Mode	Compound	*p*-Value	*q*-Value ^a^	Effect Size ^b^	Pairwise Comparison	BL-SR ^c^	SR-RC ^c^	BL-RC ^c^
147.0764:0.908:p	Glutamine	5.80 × 10^−6^	3.02 × 10^−4^	0.53	BL-SR, BL-RC	1.30	1.01	1.31
	Creatine/ Oxoproline *	1.43 × 10^−4^	1.49 × 10^−2^	0.45	BL-SR, BL-RC, SR-RC	2.40	0.87	2.10
	Glutamine/ Indoxyl Sulfate	3.01 × 10^−4^	0.015	0.40	BL-SR, BL-RC	2.65	0.90	2.39
	Carnitine/ Oxoproline	1.00 × 10^−3^	0.010	0.35	BL-SR, BL-RC	1.87	0.88	1.65
	Creatine/ Indoxyl Sulfate *	1.42 × 10^−3^	0.015	0.42	BL-SR, BL-RC	2.70	0.82	2.21
	Carnitine/ Indoxyl Sulfate	2.34 × 10^−3^	0.015	0.32	BL-SR, BL-RC	2.13	0.86	1.82
162.1125:0.666:p	Carnitine (C0)	2.53 × 10^−3^	0.019	0.31	BL-SR, SR-RC	1.21	0.87	1.05
132.0768:0.710:p	Creatine *	0.010	0.019	0.34	BL-SR, SR-RC	1.57	0.80	1.26
212.0023:1.025:n	Indoxyl sulfate	0.021	0.12	0.21	BL-SR, BL-RC	0.67	1.04	0.69
128.0353:1.025:n	Oxoproline	0.025	0.13	0.20	BL-SR	0.67	1.09	0.73
150.0583:0.890:p	Methionine *	0.028	0.13	0.21	BL-SR	1.20	0.93	1.11
178.051:0.880:n	Hippuric acid	0.033	0.14	0.19	BL-RC	0.71	0.96	0.68
146.1176:0.615:p	Deoxycarnitine *	0.044	0.17	0.19	BL-SR	1.16	0.96	1.12

mode: p = positive mode, n = negative mode; * adjusted for sex; ^a^ p-adjusted value (q-value) based on False Discovery Rate (FDR) using Benjamini-Hochberg procedure; ^b^ Effect size measured using Partial Eta Square; ^c^ Mean fold-change ratio when comparing relative ion response ratio for metabolite between two time points.

## Supplementary Materials

The following are available online at https://www.mdpi.com/2218-1989/9/7/134/s1, Table S1: Summary of 47 plasma metabolites consistently detected in a majority of samples with adequate precision when using MSI-CE-MS. Figure S1: Summary of changes in fasting plasma glucose and oral glucose tolerance (2hPG) responses in terms of (A) mM and (B) mg/dL following step reduction and recovery in a cohort of overweight, pre-diabetic older adults. Figure S2: Metabolic trajectories of the top-ranked 8 plasma metabolites associated with physical inactivity at baseline, step reduction and recovery. Figure S3: Ratiometric plasma markers associated with physical inactivity at baseline, step reduction and recovery. Figure S4: Correlation matrix/2D heat map of top-ranked plasma metabolites associated with physical inactivity from step reduction.
