# Supplementary material for: Metabolic Perturbations from Step Reduction in Older Persons at Risk for Sarcopenia: Plasma Biomarkers of Abrupt Changes in Physical Activity
Source: Metabolites. 2019 Jul 8;9(7):134. doi: 10.3390/metabo9070134 (PMC6680920; doi:10.3390/metabo9070134)
Supplement: Supplementary file 1 [file metabolites-09-00134-s001.pdf]

SUPPORTING INFORMATION

Metabolic Perturbations from Step Reduction in Older Persons at Risk for Sarcopenia: Plasma Biomarkers of Abrupt Changes in Physical Activity

Michelle Saoi,[1] Alice Li,[1] Chris McGlory,[2] Tanner Stokes,[2] Mark T. von Allmen,[2] Stuart M. Phillips,[2] Philip Britz-McKibbin[1]*

[1] Department of Chemistry and Chemical Biology, McMaster University, Hamilton, Canada

[2]Department of Kinesiology, McMaster University, Hamilton, Canada

Corresponding author*: Philip Britz-McKibbin (E-mail: britz@mcmaster.ca)

Supplemental Information: Table S1, Figure S1, Figure S2, Figure S3, Figure S4

Table S1: Summary of 47 plasma metabolites consistently detected in a majority (> 75%) of samples with adequate precision (QCs with CV < 30%, *n*=25) in this step reduction intervention study by MSI-CE-MS.

m/z:RMT:mode	Formula	Compound ID	Mass error (ppm)	Classification	CV%
67.0720:0.458:p	--	Unknown $[M+H]^+$	16.4	--	15.4
76.0393:0.643:p	$C_2H_5NO_2$	Glycine	11.3	Amino acid	15.0
90.0550:0.716:p	$C_3H_7NO_2$	Alanine	5.3	Amino acid	16.7
104.0706:0.776:p	$C_4H_9NO_2$	3-Aminoisobutyric acid (BAIBA)	5.1	Amino acid	16.6
104.1075:0.462:p	$C_5H_{14}NO$	Choline	6.0	Quaternary ammonium salt	24.8
106.0499:0.826:p	$C_3H_7NO_3$	Serine	1.2	Amino acid	15.3
114.0662:0.522:p	$C_4H_7N_3O$	Creatinine	-0.8	Amino acid	20.2
116.0706:0.903:p	$C_5H_9NO_2$	Proline	-1.8	Amino acid	12.5
118.0863:0.814:p	$C_5H_{11}NO_2$	Valine	-1.6	Amino acid	18.9
118.0863:0.963:p	$C_5H_{11}NO_2$	Betaine	-0.7	Amino acid	30.5
120.0655:0.878:p	$C_4H_9NO_3$	Threonine	-4.3	Amino acid	12.4
132.0656:1.023:p	$C_5H_9NO_3$	Hydroxyproline	0.9	Amino acid derivative	16.9
132.0768:0.710:p	$C_4H_9N_3O_2$	Creatine	-2.2	Amino acid derivative	20.9
132.1019:0.831:p	$C_6H_{13}NO_2$	Isoleucine	-2.3	Amino acid	16.3
132.1019:0.843:p	$C_6H_{13}NO_2$	Leucine	-2.1	Amino acid	15.3
133.0608:0.878:p	$C_4H_8N_2O_3$	Asparagine	-17.3	Amino acid	12.5
133.0969:0.469:p	$C_5H_{12}N_2O_2$	Ornithine	0.1	Amino acid	19.2
134.0488:0.980:p	$C_4H_7NO_4$	Aspartic Acid	-13.8	Amino acid	14.8
137.0457:1.096:p	$C_5H_4N_4O$	Hypoxanthine	-1.6	Purine derivative	19.9
144.0988:0.971:p	$C_7H_{13}NO_2$	Proline betaine	-2.3	Amino acid derivative	20.5
146.1176:0.615:p	$C_7H_{16}NO_2$	Deoxycarnitine	9.9	Amino acid derivative	17.1
147.0764:0.908:p	$C_5H_{10}N_2O_3$	Glutamine	-2.8	Amino acid	12.4
147.1128:0.472:p	$C_6H_{14}N_2O_2$	Lysine	-2.7	Amino acid	25.0
148.0604:0.924:p	$C_5H_9NO_4$	Glutamic Acid	-2.9	Amino acid	20.2
150.0583:0.890:p	$C_5H_{11}NO_2S$	Methionine	1.6	Amino acid	21.1
156.0768:0.530:p	$C_6H_9N_3O_2$	Histidine	-4.4	Amino acid	25.8
160.1331:0.653:p	$C_8H_{17}NO_2$	Unknown $[M+H]^+$	16.2	--	15.9
161.1285:0.496:p	$C_7H_{16}N_2O_2$	Unknown $[M+H]^+$	0.6	--	20.5
162.1125:0.666:p	$C_7H_{15}NO_3$	Carnitine	-2.3	Amino acid derivative	15.1

166.0863:0.925:p	$C_9H_{11}NO_2$	Phenylalanine	16.1	Amino acid	19.1
170.0924:0.551:p	$C_7H_{11}N_3O_2$	3-Methylhistidine	-3.9	Amino acid derivative	15.9
175.1190:0.503:p	$C_6H_{14}N_4O_2$	Arginine	-1.6	Amino acid	23.5
176.1030:0.938:p	$C_6H_{13}N_3O_3$	Citrulline	-1.1	Amino acid derivative	13.0
182.0812:0.962:p	$C_9H_{11}NO_3$	Tyrosine	-2.0	Amino acid	13.4
204.1230:0.734:p	$C_9H_{17}NO_4$	Acetylcarnitine	1.3	Acylcarnitine	19.5
276.1185:1.125:p	$C_{10}H_{17}N_3O_6$	Unknown $[M+H]^+$	-2.4	*NA*	13.2
89.0244:1.186:n	$C_3H_6O_3$	Lactic acid	5.8	Organic acid	6.1
101.0608:0.983:n	$C_5H_{10}O_2$	Isovaleric acid	-3.3	Fatty acid derivative	10.9
102.0510:0.936:n	$C_4H_9NO_2$	Dimethylglycine	0.5	Amino acid derivative	12.1
117.0557:0.988:n	$C_5H_{10}O_3$	2-Hydroxyvaleric acid	-3.0	Fatty acid derivative	25.4
128.0353:1.025:n	$C_5H_7NO_3$	Oxoproline	-1.2	Amino acid derivative	8.2
167.0211:0.947:n	$C_5H_4N_4O_3$	Uric acid	-1.0	Purine derivative	7.0
178.0510:0.880:n	$C_9H_9NO_3$	Hippuric acid	2.6	Organic acid	12.3
179.0564:0.455:n	$C_6H_{12}O_6$	Glucose	2.9	Monosaccharide	7.1
187.0071:1.082:n	$C_7H_8O_4S$	p-Cresol sulfate	-3.8	Phenylsulfate	23.8
212.0023:1.025:n	$C_8H_7NO_4S$	Indoxyl sulfate	20.4	Phenylsulfate	31.1
263.1037:0.759:n	$C_{13}H_{16}N_2O_4$	Phenylacetylglutamine	-1.9	Amino acid derivative	15.9

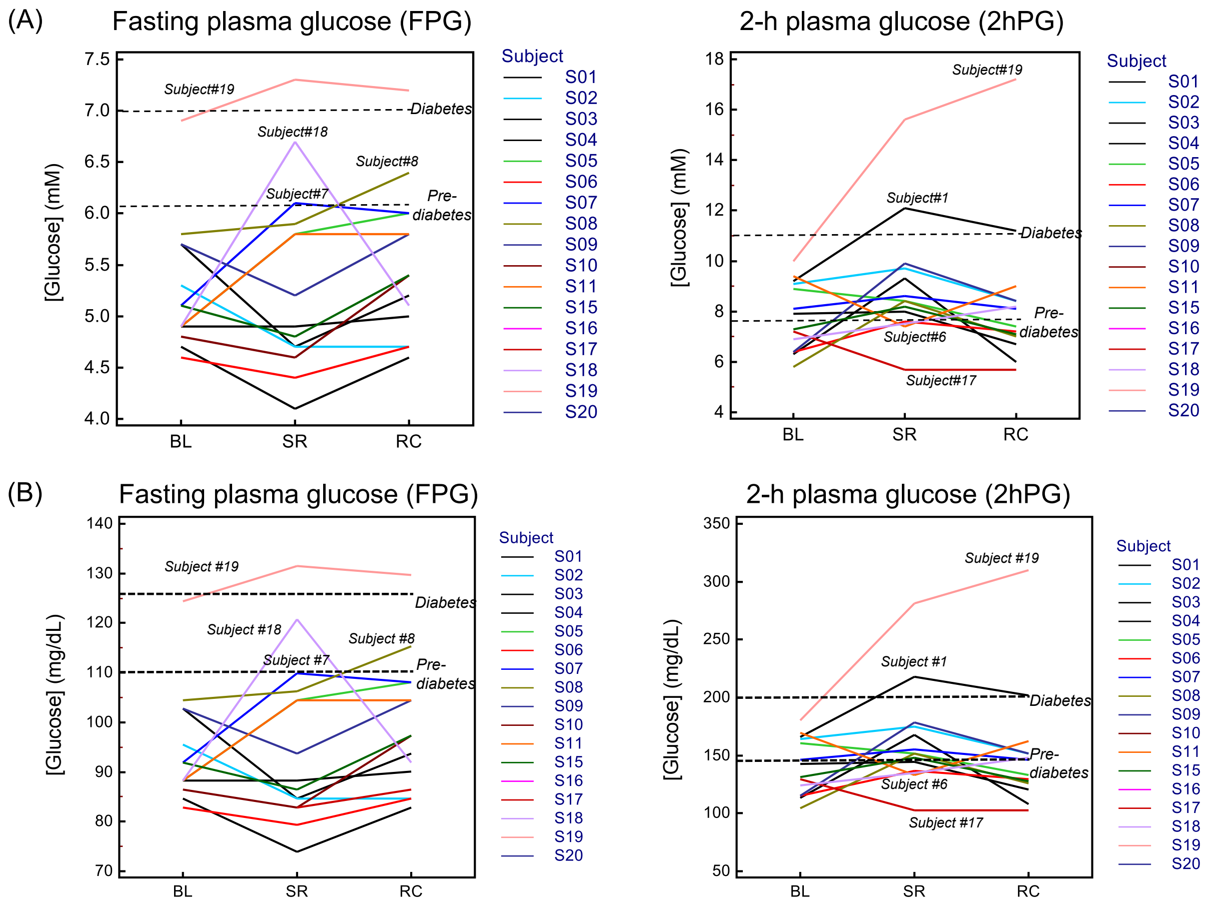

Figure S1. Summary of changes in fasting plasma glucose and oral glucose tolerance (2hPG) responses in terms of (A) mM and (B) mg/dL following step reduction and recovery in a cohort of overweight and largely pre-diabetic participants. Overall, 7 (of 15 or 47%) participants were defined as pre-diabetic with impaired glucose tolerance (7.8-11 mM or 140-199 mg/dL 2hPG) with one subject also having impaired fasting glucose at baseline (BL). However, following the step reduction (SR) and/or the recovery (RC) period, most participants in this cohort were defined as pre-diabetic (12 out of 15 or 80%) with one participant becoming diabetic with impaired fasting glucose (> 7.0 mM or 126 mg/dL) and grossly elevated 2hPG (> 11.1 mM or 200 mg/dL) based on diagnostic criteria defined by Diabetes Canada. Overall, there were only two participants in this cohort (among 15 with blood glucose measurements) who maintained normal glucose homeostasis and glucose tolerance throughout the intervention period.

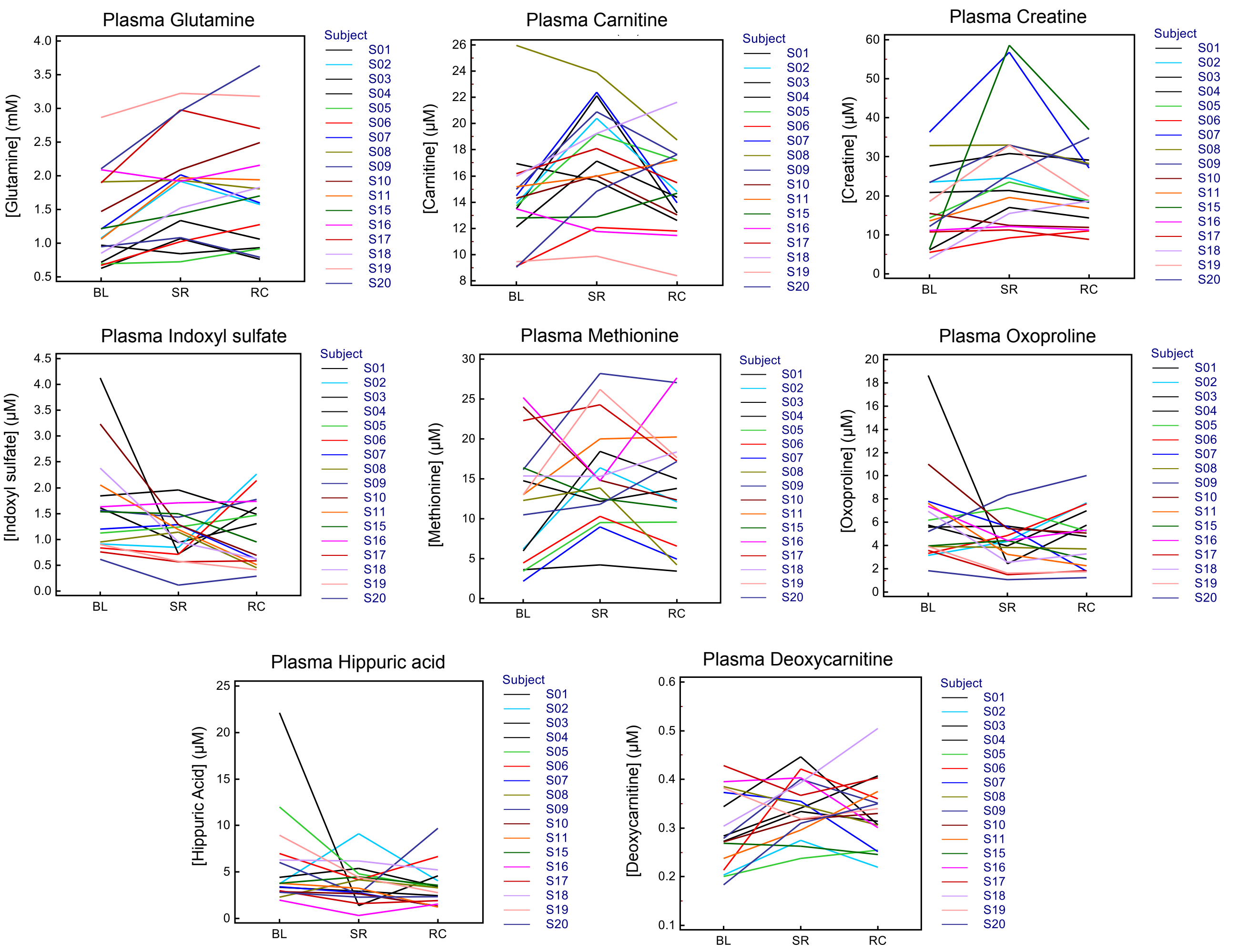

Figure S2. Individual metabolic trajectories for the top-ranked 8 plasma metabolites associated with physical inactivity from a cohort of overweight/pre-diabetic older adults at baseline (BL), following 2 weeks of step reduction (SR) and after resuming normal habitual activity upon recovery for two weeks (RC). Despite collection of blood specimens under standardized conditions with fasting/morning plasma samples collected after participants were provided 2 days of standardized meals, there was considerable between-subject variability in circulating plasma metabolite concentrations, including their dynamic responses to step reduction.

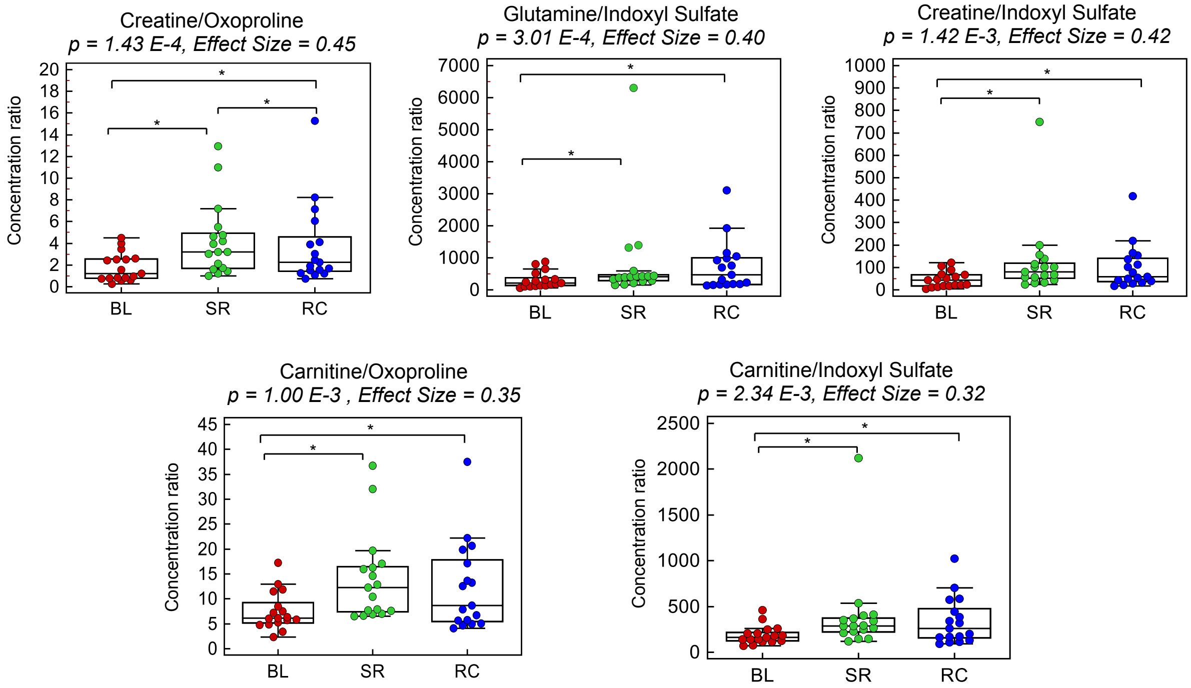

Figure S3. Ratiometric plasma markers associated with physical inactivity from a cohort of overweight/pre-diabetic older adults at baseline (BL), following 2 weeks of step reduction (SR) and after resuming normal habitual activity upon recovery for two weeks (RC). All ratiometric biomarker satisfied Benjamini-Hochberg adjustment ($q < 0.05$, FDR) for multiple hypothesis testing, which increased the effect size as compared to most single plasma metabolites (*e.g.,* creatine, oxo-proline, indoxyl sulfate) with the exception of glutamine and carnitine. Overall, most ratiometric markers reflected significant changes in circulating metabolites associated with muscle tissue energy metabolism and oxidative stress upon step reduction with a persistent perturbation from baseline even after two weeks of recovery with normal habitual activity.

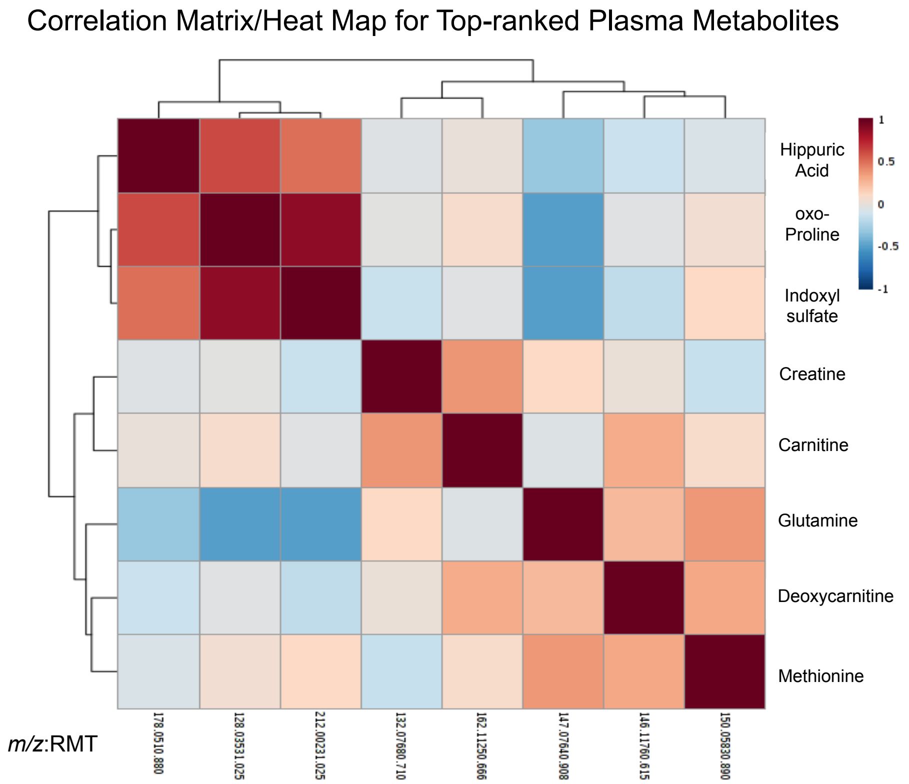

Figure S4. A correlation matrix/2D heat map among the top-ranked plasma metabolites (*glog*-transformed ion responses) identified in this step reduction intervention study, which highlights the strong co-linearity between circulating levels of indoxyl sulfate and oxo-proline (r = 0.881; $p = 1.0\times10^{-15}$), hippuric acid and oxo-proline (r = 0.622; $p = 1.1\times10^{-6}$), as well as hippuric acid and indoxyl sulfate (r = 0.507; $p = 1.4\times10^{-4}$). Additionally, there was an inverse correlation between glutamine and oxoproline (r = -0.502; $p = 1.8\times10^{-4}$), as well as glutamine and indoxyl sulfate (r = -0.500; $p = 1.8\times10^{-4}$) suggesting common metabolic pathways involved in their regulation in circulation.
